# Tolerance and Antioxidant Activity of Watermelon Cultivars Pre-Treated with Stress Attenuators and Subjected to Water Deficit

**DOI:** 10.3390/plants15020184

**Published:** 2026-01-07

**Authors:** Moadir de Sousa Leite, Salvador Barros Torres, Clarisse Pereira Benedito, Kleane Targino Oliveira Pereira, Maria Valdiglezia de Mesquita Arruda, Roseane Rodrigues de Oliveira, Giovanna Dias de Sousa, Cynthia Cavalcanti de Albuquerque, Marciana Bizerra de Morais, Charline Zaratin Alves, Givanildo Zildo da Silva, Emerson de Medeiros Sousa, Pablo Ferreira da Silva, Cibele Chalita Martins, Francisco Vanies da Silva Sá

**Affiliations:** 1 Department of Agricultural and Forestry Sciences, Federal Rural University of the Semi-Arid, Mossoró 59625-900, RN, Brazil; moadir@outlook.com (M.d.S.L.); sbtorres@ufersa.edu.br (S.B.T.); clarisse@ufersa.edu.br (C.P.B.); kleane_rn@hotmail.com (K.T.O.P.); valdigleziaarruda@yahoo.com.br (M.V.d.M.A.); roseaneoliveira11@hotmail.com (R.R.d.O.); giodiassousa@hotmail.com (G.D.d.S.); pablo.silva02405@alunos.ufersa.edu.br (P.F.d.S.); 2 Department of Biological Sciences, Faculty of Exact and Natural Sciences, Universidade Estadual do Rio Grande do Norte, Mossoró 59610-090, RN, Brazil; cynthiacavalcanti@uern.br (C.C.d.A.); marciana.bio@gmail.com (M.B.d.M.); 3 Federal University of Mato Grosso do Sul, Chapadão do Sul 79560-000, MS, Brazil; charline.alves@ufms.br; 4 University of Rio Verde, AC Rio Verde, Setor Central, Rio Verde 75901-970, GO, Brazil; givanildo@unirv.edu.br; 5 Instituto Federal do Rio Grande do Norte, Campus Parelhas, Parelhas 59360-000, RN, Brazil; emerson.sousa@ifrn.edu.br; 6 Departament of Plant Production, Júlio de Mesquita Filho, São Paulo University, Jaboticabal 14884-900, SP, Brazil; cibele.chalita@unesp.br; 7 Department of Agrarian and Exact Sciences, Universidade Estadual da Paraíba, Sítio Cajueiro, Catolé Do Rocha 54888-000, PB, Brazil

**Keywords:** abiotic stress, *Citrullus lanatus*, Cucurbitaceae, water deficit, oxidative stress

## Abstract

This study aimed to evaluate the effect of stress attenuators on the tolerance and antioxidant activity of watermelon cultivars under water deficit. The experiment was conducted in two stages, Stage I corresponding to water deficit levels (N1 = 0; N2 = −0.1; N3 = −0.2 MPa) and six watermelon cultivars. Stage II comprises two cultivars selected in Stage I (one sensitive and one tolerant) and the combination of water restriction with attenuators (T1 = 0.0 MPa (control), T2 = −0.2 MPa (water deficit), T3 = −0.2 MPa + hydropriming, T4 = −0.2 MPa + gibberellic acid, T5 = −0.2 MPa + salicylic acid, and T6 = −0.2 MPa + hydrogen peroxide). The concentration and exposure times of the attenuators were determined through preliminary tests. In Stage I, physiological and biochemical analyses were performed. In Stage II, in addition to these tests, hydrogen peroxide content, malondialdehyde levels, and the activity of superoxide dismutase (SOD), catalase (CAT), and ascorbate peroxidase (APX) were assessed. Water deficit impaired germination and seedling vigor of watermelon, with Crimson Sweet, Omaru, Charleston Gray, and Congo being the most sensitive cultivars, while Fairfax was the most tolerant. For Crimson Sweet, pre-germination treatments reduced oxidative stress and enhanced tolerance by stimulating antioxidant enzyme activity, with GA and H_2_O_2_ providing the most effective results. For Fairfax, greater tolerance was associated with osmotic adjustment through the accumulation of compatible solutes, a mechanism further enhanced by the use of attenuators.

## 1. Introduction

Plants inhabit dynamic environments that are often unfavorable or stressful for growth and development. Among adverse environmental conditions, water deficit is one of the main factors affecting the geographic distribution of plants, limiting productivity, and threatening food security [1]. In this context, it is essential to understand how water limitation affects agriculturally important species, as it has profound implications for crop performance and resilience.

Watermelon [*Citrullus lanatus* (Thunb.) Matsum & Nakai.] is the most important cucurbit in terms of global production volume. This vegetable is widely consumed worldwide due to its fruits, which are nutritious and rich in natural sugars, amino acids, lycopene, organic acids, and essential nutrients [2]. Brazil is currently the fourth-largest watermelon producer in the world [3], with production concentrated in the Northeast region, where most cultivation areas are located in the semi-arid zone, where water scarcity significantly affects plant growth and productivity [4].

In this context, the negative effects of water deficit are particularly severe during germination and the early stages of growth, which represent the most vulnerable phases of the plant life cycle, including *Cucurbita* species [4,5], and may be aggravated by the combination of limited water availability and high temperatures [6]. However, the severity of these impacts also depends on the intensity and duration of water scarcity and, above all, on the genetic characteristics of each genotype [7].

Thus, one of the main consequences of water deficit in plants is oxidative stress, resulting from the production of reactive oxygen species (ROS). These molecules are products of oxidation–reduction reactions that occur naturally in plant metabolism; however, under stress conditions, their accumulation intensifies, leading to redox imbalance and cellular damage [8]. To maintain homeostasis, plants rely on both enzymatic and non-enzymatic biochemical mechanisms. Non-enzymatic defenses involve the accumulation of compatible solutes, whereas enzymatic defenses constitute the main system responsible for eliminating excess ROS. In this sense, the activity of antioxidant enzymes is directly associated with tolerance to water deficit [9].

Given their great importance, studies seek to enhance this response. Seed pre-germinative treatments have been employed as a potential strategy to mitigate the effects of abiotic stress and improve plant adaptability [10]. In this context, defense responses are enhanced by phytohormones, which modulate antioxidant signaling and responses, thereby increasing the expression and activity of enzymes that eliminate ROS.

This response is evidenced in several studies, which have demonstrated the benefits of seed pre-germinative treatments on watermelon germination and seedling establishment, including halopriming and hydropriming [11]; gibberellic and salicylic acid treatments in melon [12]; ascorbic acid in cucumber [13]; and hydrogen peroxide in watermelon [14]. However, these studies did not clarify how these attenuators modulate tolerance to water deficit in watermelon, especially with respect to the antioxidant mechanisms involved.

Understanding the complexity of plant defense responses under drought stress arises from the lack of a uniform modulation of resistance mechanisms, which vary between morphophysiological and biochemical processes [9,13]. In this regard, strategies involving selection, breeding, and molecular and/or biochemical studies may enable marker-assisted selection, facilitating the development of plants capable of sustaining yield even under adverse conditions.

From this perspective, the hypothesis of this study is that attenuators may improve watermelon seed germination and vigor under water deficit, with distinct metabolic responses in sensitive and tolerant genotypes. Therefore, this work aimed to evaluate the effect of stress attenuators on tolerance, germination, osmotic adjustment, and antioxidant activity in watermelon cultivars under water deficit.

## 2. Results

### 2.1. Stage I

The analysis of variance revealed a significant interaction between cultivars and water restriction levels for all evaluated variables (Table 1).

**Figure 1:**
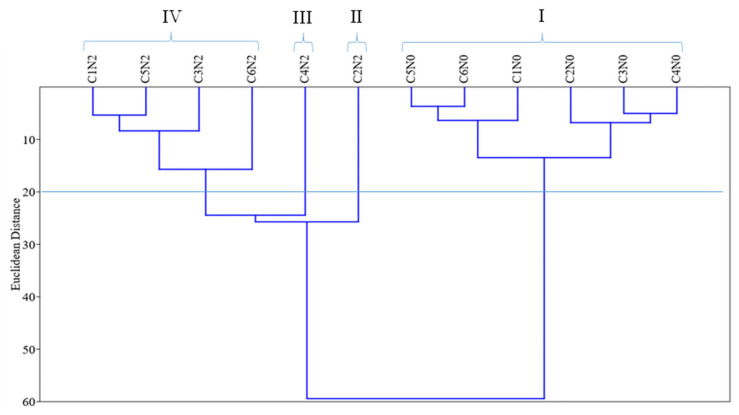
Dissimilarity dendrogram of groups formed by combining water restriction levels (N) and watermelon cultivars (C). N0 = control; N2 = −0.2 MPa; C1 = Crimson Sweet; C2 = Fairfax; C3 = Charleston Gray; C4 = Charleston Super; C5 = Omaru; C6 = Congo.

Overall, the results showed that the cultivars Crimson Sweet and Fairfax stood out as the most sensitive and tolerant to water deficit, respectively, a finding also confirmed by the dissimilarity dendrogram (Figure 1). The Crimson Sweet susceptibility was mainly characterized by reductions in germination and seedling growth, which were significant even at the lowest stress level, and by a limited capacity to regulate osmotic potential, as confirmed by the biochemical variables. In contrast, Fairfax exhibited tolerance to water deficit, characterized by its efficient osmotic adjustment and higher accumulation of total soluble sugars, free amino acids, and proline (Table 1).

When selecting the water deficit level to apply in the second stage, emphasis was placed on reductions in germination and seedling performance traits. Accordingly, the level of −0.2 MPa was adopted, as it caused significant impairment in both the sensitive and tolerant cultivars while still allowing the possibility of recovery of germination, growth, and dry mass through the use of attenuators in the second stage.

Cluster analysis (Figure 1), based on Euclidean distance with a cutoff of 20.0, identified four groups formed by combinations of osmotic potential levels (N) and watermelon cultivars (C). Group I comprised all six cultivars under control conditions (0.0 MPa).

Groups II, III, and IV included the six cultivars under water deficit conditions. Among them, Fairfax exhibited the highest germination and biochemical and growth performance at −0.2 MPa, forming Group II, located adjacent to the control group in the dendrogram. Conversely, Group IV comprised the most sensitive cultivars, with Crimson Sweet standing out as the most sensitive, positioned farthest to the left in the dendrogram (furthest from Group I). Based on these results, −0.2 MPa was selected as the stress level for the second stage, with Fairfax and Crimson Sweet chosen as tolerant and sensitive cultivars, respectively.

### 2.2. Stage II

Analysis of variance indicated a significant interaction for all variables except root dry mass and hydrogen peroxide, for which the effects of cultivars and treatments were significant only in isolation. Thus, the effect of treatments on most variables was directly associated with the cultivar’s tolerance to water deficit.

**Figure 2:**
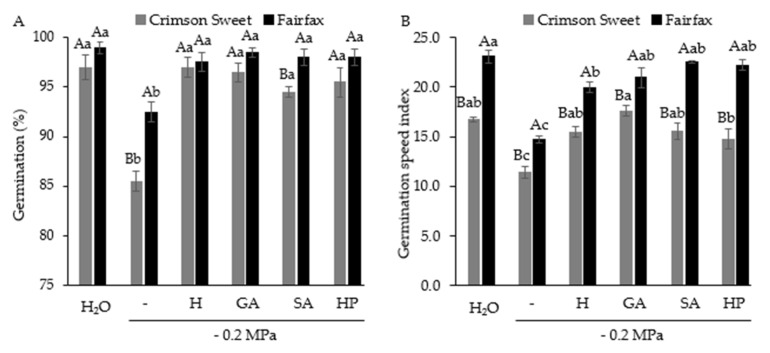
Germination (**A**) and germination speed index (**B**) of watermelon cultivars subjected to pregerminative treatments and water deficit. Control (H_2_O); water deficit without attenuators (–); water deficit + hydropriming (H); water deficit + gibberellic acid (GA); water deficit + salicylic acid (SA); water deficit + hydrogen peroxide (HP). Means followed by the same uppercase letter (cultivars) and lowercase letter (pregerminative treatments) do not differ from each other according to Student’s *t*-test and Tukey’s test, respectively, at 5% probability.

Seed germination was negatively affected by water deficit, with the greatest reduction observed in Crimson Sweet, which decreased by 12% (Figure 2A). Overall, pregerminative treatments improved germination under water-deficit conditions, with no statistical difference from the control.

Similarly to germination, the germination speed index was also reduced under water deficit, decreasing by 32% and 36% for Crimson Sweet and Fairfax, respectively, compared with the control (Figure 2B). Pregerminative treatments enhanced germination speed in both cultivars. The best results for Crimson Sweet were obtained with GA, while Fairfax responded positively to GA, SA, and HP.

**Figure 3:**
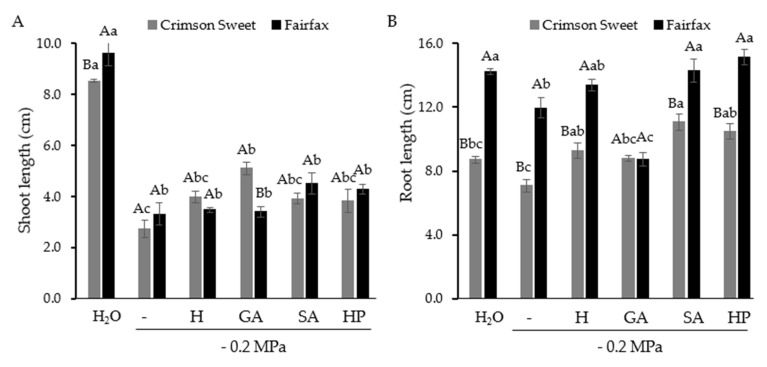
Shoot (**A**) and root (**B**) length of watermelon cultivars subjected to pregerminative treatments and water deficit.

Shoot length in both cultivars was significantly reduced by water deficit (Figure 3A). For Fairfax, pregerminative treatments did not provide significant recovery, with water deficit causing a 65% reduction compared with the control. In Crimson Sweet, seed pretreatment with GA increased shoot length by 86% compared with the water deficit condition; however, this value remained 40% lower than that of the control.

Root length was also significantly reduced by water deficit in both cultivars (Figure 3B). Pregerminative treatments generally improved root length, except for GA in Fairfax, which resulted in even lower values than under water deficit.

**Figure 4:**
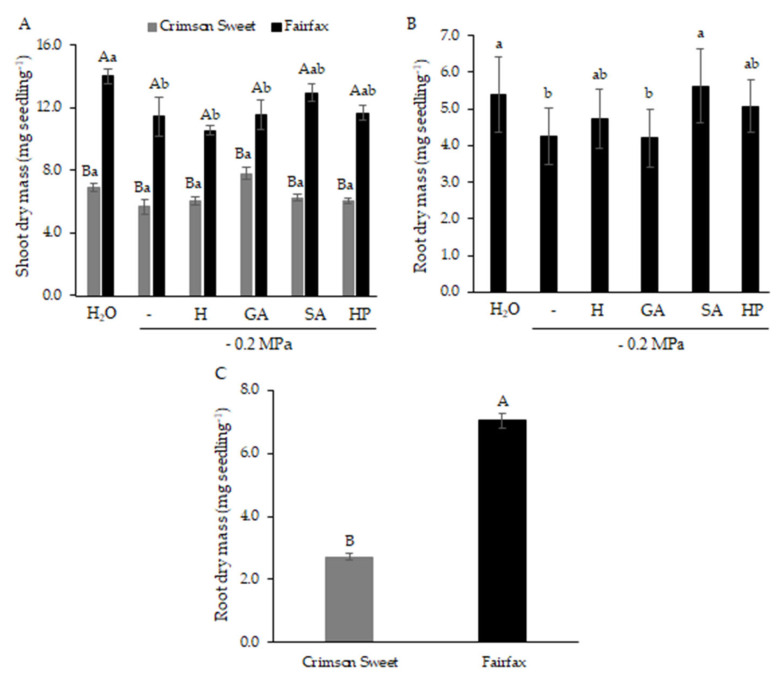
Shoot dry mass (**A**) and root dry mass (**B**) and (**C**) of watermelon cultivars subjected to pregerminative treatments and water deficit.

Although shoot dry mass in Crimson Sweet was not affected by water deficit, it remained consistently lower than that of Fairfax across all treatments (Figure 4A). In Fairfax, water deficit caused an 18% reduction compared with the control. Treatment with SA restored shoot dry mass to values statistically equal to those of the control, although not different from the water deficit condition.

Root dry mass was enhanced by attenuator treatments, except for GA, which showed no benefit compared with water deficit (Figure 4B). Pretreatment with H, HP, and SA restored root dry mass to levels similar to the control, with SA producing the highest effect, 33% greater than under water deficit. Among cultivars, Fairfax consistently outperformed Crimson Sweet, with root dry mass 2.6 times higher (Figure 4C).

**Figure 5:**
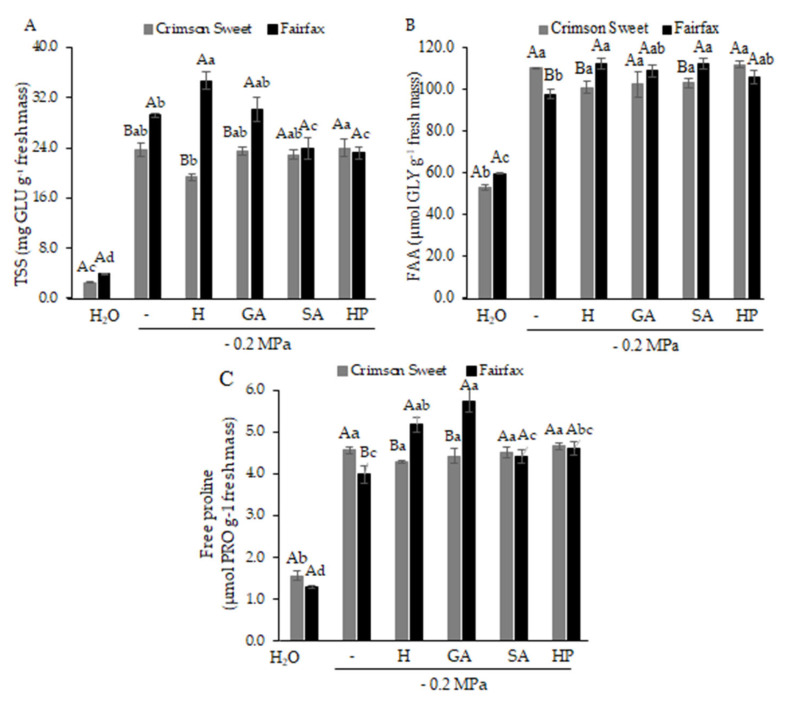
Total soluble sugars—TSS (**A**), free amino acids—FAA (**B**), and free proline (**C**) of watermelon cultivars subjected to pregerminative treatments and water deficit.

The accumulation of soluble sugars increased in response to water deficit, reaching values 9.5 and 7.3 times higher than those of the control in Crimson Sweet and Fairfax, respectively (Figure 5A). In Crimson Sweet, pregerminative treatments did not promote greater sugar accumulation than water deficit alone. In Fairfax, however, hydropriming and GA pretreatments increased soluble sugar accumulation by 18.7% and 1.0%, respectively, compared with water deficit.

Free amino acids (Figure 5B) and free proline (Figure 5C) in Crimson Sweet showed similar responses, with significant increases under water deficit. However, pregerminative treatments did not further enhance amino acid or proline accumulation compared with water deficit alone (0.0). In Fairfax, increases in free amino acids were observed for all pregerminative treatments, with the highest concentrations recorded under H and SA, although not statistically different from GA and HP. Similarly, greater proline accumulation was detected in Fairfax under H and GA pretreatments, with concentrations 30% and 44% higher, respectively, than those observed under water deficit (0.0).

**Figure 6:**
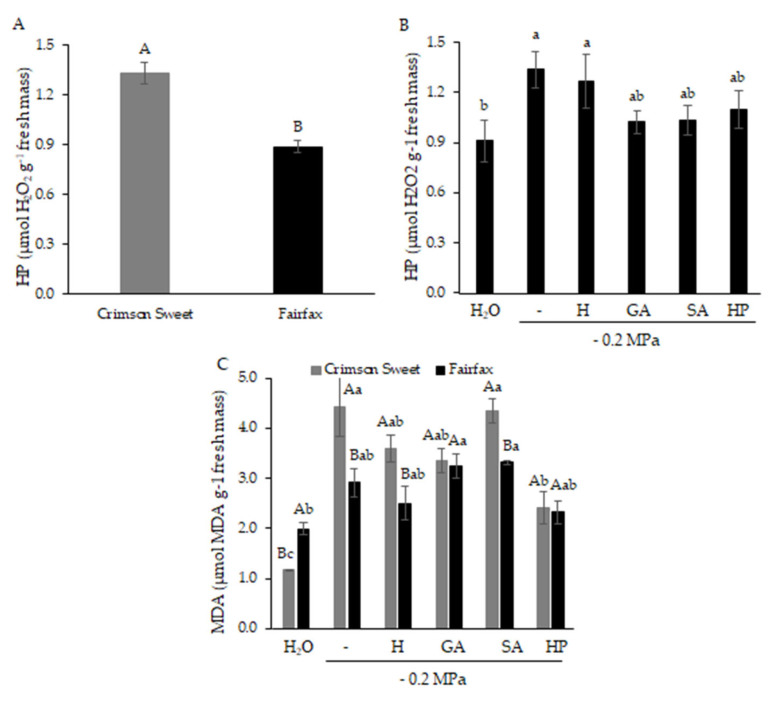
Lipid peroxidation expressed by hydrogen peroxide (**A**) and (**B**) and malondialdehyde (**C**) levels in watermelon cultivars subjected to pregerminative treatments and water deficit.

Regarding hydrogen peroxide accumulation (Figure 6A), Crimson Sweet exhibited higher concentrations, 44% higher than those of Fairfax. Hydrogen peroxide content was also influenced by water deficit (Figure 6B), with higher levels under stress. Nevertheless, pretreatments with GA, SA, and HP reduced hydrogen peroxide levels by 23%, 23%, and 15%, respectively, compared with water deficit (0.0 MPa).

Water deficit also increased malondialdehyde (MDA) levels in both cultivars (Figure 6C). In Fairfax, the lowest MDA concentrations were recorded under H and HP, although these values were not statistically different from water deficit (0.0). In Crimson Sweet, H, GA, and HP pretreatments reduced MDA content by 18%, 23%, and 45%, respectively, compared with water deficit (0.0).

**Figure 7:**
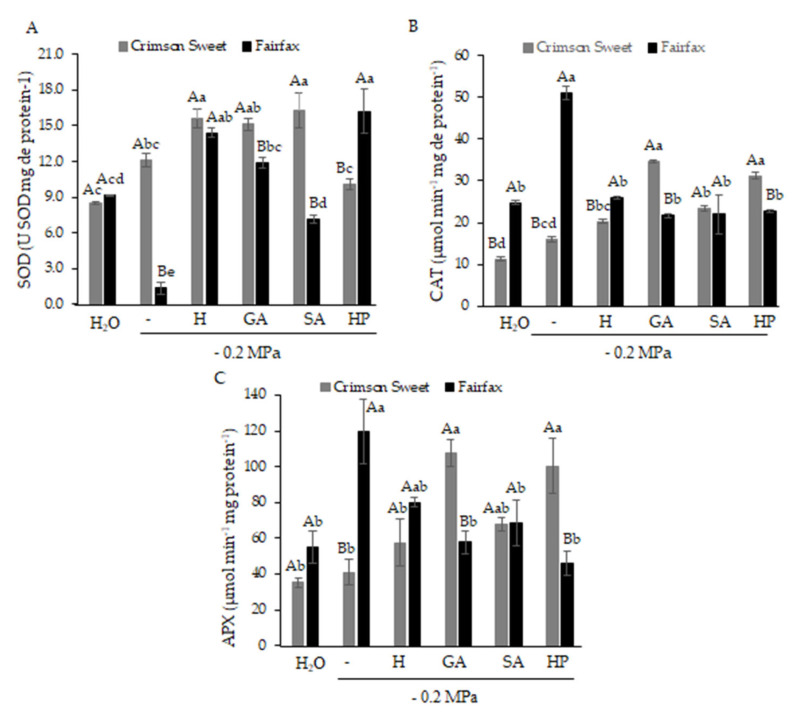
Activity of superoxide dismutase—SOD (**A**), catalase—CAT (**B**), and ascorbate peroxidase—APX (**C**) in watermelon cultivars subjected to pregerminative treatments and water deficit.

Enzymatic antioxidant activity was significantly affected by both water deficit and seed pretreatment. In Crimson Sweet, pregerminative treatments significantly increased the activities of superoxide dismutase (SOD) (Figure 7A), catalase (CAT) (Figure 7B), and ascorbate peroxidase (APX) (Figure 7C). Compared with water deficit (0.0), SOD activity increased markedly under H (29%), GA (25%), and SA (35%). For CAT and APX, the highest increases were obtained with GA (115% and 163%) and HP (94% and 145%), respectively.

The antioxidant defense system of Fairfax behaved differently from that of Crimson Sweet, which may be associated with its higher tolerance to water deficit. Under water deficit alone (0.0), SOD activity was nearly undetectable, whereas CAT and APX activities were higher than in all other treatments, being 97% and 50% greater, respectively, than under H, the attenuator that most stimulated enzyme activity.

## 3. Discussion

**Figure 8:**
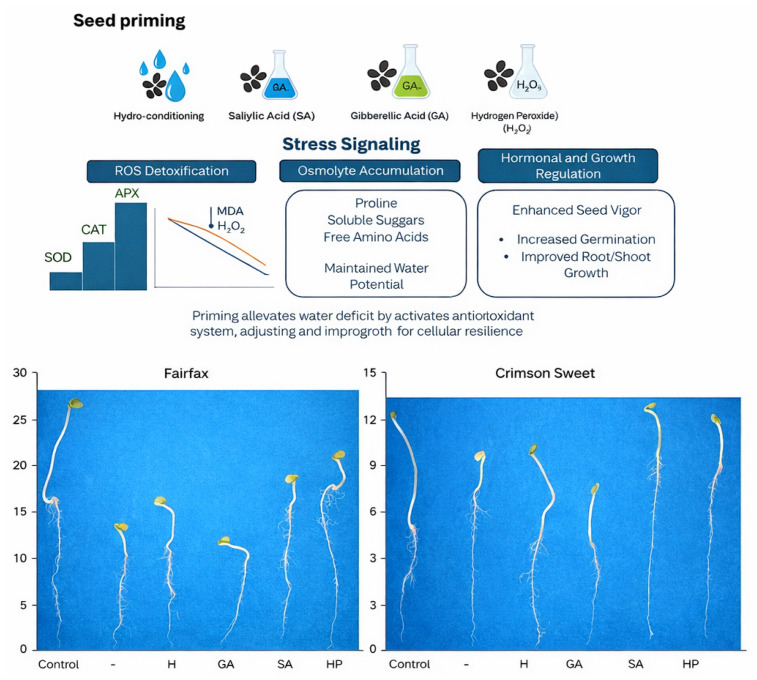
Proposed mechanisms of tolerance in watermelon by seed priming.

Pre-germinative treatments primarily promote seed germination and seedling uniformity [15]. These benefits result from the stimulation of DNA repair mechanisms and the activation of pre-germinative metabolism, which increase enzymatic activity and mobilize reserves [16]. Such treatments are therefore important techniques for enhancing tolerance to abiotic and biotic stresses during germination, one of the most sensitive stages in the plant life cycle (Figure 8).

A reduced germination rate under water deficit is widely reported in the literature and is attributed to delayed tissue rehydration and subsequent metabolic events, which are entirely dependent on water availability [17]. In this context, the use of plant growth regulators and techniques that induce and standardize seed germination is highly relevant. Among germination inducers, GA is notable for its role in the synthesis of hydrolytic enzymes, particularly α-amylases, which degrade polysaccharides into monosaccharides, facilitating their metabolism and subsequent energy production during germination [18].

In addition to reducing germination speed and rate, water deficit also negatively affects early seedling development. Similar findings were reported by [19], who observed reduced cucumber seedling length under water stress. However, in the present study, watermelon seedlings displayed differential sensitivity between shoots and roots, with roots being less affected by water deficit. Biomass partitioning can shift toward greater root development under water scarcity [20], resulting in a reduced shoot-to-root ratio. This adjustment constitutes an important strategy for ensuring water uptake under drought conditions [21].

Changes in early seedling growth and dry mass accumulation also result from the use of stress-attenuating agents, with responses directly linked to the defense pathways activated by each regulator. SA acts directly on the antioxidant system by stimulating the synthesis and activity of enzymatic defense compounds under stress [22]. At low concentrations, H_2_O_2_ not only positively influences germination but also stimulates the antioxidant system and regulates the interaction between redox status and plant hormones, inducing proteins involved in signaling and early plant development [23]. GA promotes cell division and expansion, increasing seedling length [24]. However, GA may also reduce root length [25], as enhanced cell division and elongation in shoot buds favor aerial growth while inhibiting or suppressing root development, as observed in cv. Crimson Sweet.

The accumulation of osmoprotectants under water deficit represents another key tolerance mechanism [26]. These compatible solutes maintain cellular water balance without disrupting normal physiological processes. Increased levels of sugars and proline, for example, protect macromolecules such as proteins, nucleic acids, lipids, and membranes from oxidative damage [27]. Thus, the higher accumulation of compatible solutes in Fairfax seeds subjected to pre-germinative treatments proved critical for improving drought tolerance, since osmotic adjustment via solute accumulation significantly contributed to stress defense, reducing plant water potential and favoring water uptake.

Under water stress, plants increase the production of reactive oxygen species (ROS), such as hydrogen peroxide (H_2_O_2_), across different cellular compartments [8]. In general, ROS are by-products of redox reactions arising from changes in electron distribution during metabolic processes such as photorespiration and photosynthesis [28]. If uncontrolled, ROS levels rise within cells and cause oxidative damage to membranes (lipid peroxidation), proteins, RNA, and DNA, potentially leading to oxidative cell death, a condition referred to as oxidative stress [8].

The interaction of ROS with biomolecules is evidenced by increased malondialdehyde (MDA) levels, a marker of lipid peroxidation and, consequently, of oxidative membrane damage caused by H_2_O_2_ accumulation [29]. Thus, lower H_2_O_2_ and MDA contents indicate reduced damage under water deficit, either due to cultivar tolerance or the mitigating effects of the regulators, as observed in this study.

Overall, the enzymatic complex results corroborate previous findings. SOD is considered the first line of antioxidant defense against stress, catalyzing the conversion of superoxide anion (O_2_^−^) into H_2_O_2_ and O_2_, with hydrogen peroxide subsequently converted into H_2_O and O_2_ by CAT and APX [30].

The activation of the antioxidant system occurs regardless of genotype tolerance [31]. However, both the magnitude of activation and the specific enzymes involved may vary. For example, tolerant genotypes generally exhibit stronger CAT and APX activation than sensitive genotypes. Notably, in this study, the attenuators also modulated this dynamic by enhancing SOD activity in both cultivars.

Physiological priming techniques and the application of regulators such as gibberellic acid, salicylic acid, and hydrogen peroxide have been used to enhance plant responses to abiotic stresses [32]. In the present study, these approaches influenced perception, signaling, and response mechanisms in watermelon seedlings under water deficit, leading to metabolic reprogramming and enhanced expression of the defense system. Revisiting the guiding question, our findings illuminate how priming can modulate antioxidant cascades, thereby providing a comprehensive picture of priming effects and addressing the initial gap in understanding the mechanisms at play in watermelon.

Biochemical systems, both enzymatic and non-enzymatic, act in coordination to prevent ROS overproduction and maintain redox balance, with their occurrence and effectiveness depending on multiple factors [33]. In this study, differences in drought tolerance between cultivars were associated with the greater ability of cv. Fairfax accumulates compatible solutes. In this cultivar, pre-germinative treatments stimulated and enhanced the performance of these systems, promoting adequate cellular water status, mitigating stress-induced damage, and reducing the intensity of enzymatic responses.

The findings demonstrate that the distinct tolerance levels of watermelon cultivars to water deficit are related to the specific mechanisms each cultivar employs to cope with stress. Furthermore, these mechanisms were enhanced by pre-germinative seed treatments with stress-attenuating agents, offering potential benefits to farmers in Brazil’s main watermelon-producing region, which faces frequent drought. Therefore, continued research on seed treatments with attenuators is recommended, given the wide range of new techniques and compounds with potential for effective application and promising results.

## 4. Materials and Methods

The study was conducted at the Seed Analysis Laboratory (LAS) of the Department of Agronomic and Forestry Sciences (DCAF) of the Federal Rural University of the Semi-Arid (UFERSA) and at the Plant Physiology and Biochemistry Laboratory (LFBP) of the State University of Rio Grande do Norte (UERN), in Mossoró, RN, Brazil.

The experiment was divided into two stages. In the first stage, two watermelon cultivars (one sensitive and one tolerant to stress) were selected, and the level of water deficit to be applied in the second stage was determined. In the second stage, the species’ enzymatic defense system was evaluated by pre-treating seeds with attenuators and exposing them to water-deficit conditions.

### 4.1. Stage I

The experimental design was completely randomized, with four replicates of 50 seeds each, in a 3 × 6 factorial arrangement. The first factor corresponded to water deficit levels (N1 = 0; N2 = −0.1; N3 = −0.2 MPa), and the second factor consisted of six watermelon cultivars (C1 = Crimson Sweet; C2 = Fairfax; C3 = Charleston Gray; C4 = Charleston Super; C5 = Omaru; C6 = Congo).

To simulate water-deficit conditions, seeds from each replicate were germinated between three sheets of paper towel moistened with polyethylene glycol (PEG 6000) solutions at 0, −0.1, and −0.2 MPa osmotic potential [34]. Each replicate was moistened with a solution equivalent to 2.5 times the paper’s dry weight [35]. The rolls were then placed in plastic bags and incubated in a germination chamber at 25 °C, under an 8-h photoperiod, with a light intensity of approximately 200–300 lux (≈ 5–8 µmol m^−2^ s^−1^).

Evaluations were performed daily by counting germinated seeds up to the 14th day after sowing [35]. Seeds that developed normal seedlings, that is, with well-developed, complete, and undamaged essential structures, were considered germinated. The variables analyzed were:

- Germination (G): percentage of normal seedlings at 14 days after sowing [35].
- Germination speed index (GSI): calculated concurrently with the germination test, with daily counts of normal seedlings up to the 14th day, following the method proposed by Maguire [36].
- Seedling shoot length (SL) and root length (RL): at the end of the germination test, 10 normal seedlings were randomly selected, and shoot and root lengths were measured using a millimeter ruler.
- Seedling shoot dry mass (SDM) and root dry mass (RDM): the seedlings used for biometric measurements were sectioned, placed in kraft paper bags, and dried in a forced-air oven at 65 °C. They were then weighed on a precision balance (0.0001 g), with results expressed in mg per seedling.

The remaining normal seedlings from each replicate were collected, placed in Falcon tubes, frozen in liquid nitrogen (−196 °C), and stored in an ultra-freezer (−80 °C). The plant material was used to obtain crude extracts. For this purpose, 0.2 g of fresh seedling mass was placed in hermetically sealed tubes with 3 mL of 60% ethanol for automatic maceration. After maceration, the tubes were incubated in a water bath at 60 °C for 20 min and centrifuged at 10,000 rpm for 10 min at 4 °C. The supernatant was collected to determine the following variables:

- Total soluble sugars (TSS): determined using the anthrone method [37] with a glucose standard curve, results expressed as mg glucose g^−1^ fresh mass.
- Total free amino acids (TFAA): determined by the acid-ninhydrin method [38] using a glycine standard curve; results expressed as mg glycine g^−1^ fresh mass.
- Free proline (FP): quantified using the method proposed by Bates et al. [39], results expressed as µmol proline g^−1^ fresh mass.

### 4.2. Stage II

The second stage was conducted in a 2 × 6 factorial design. The first factor consisted of the two cultivars selected in Stage I (one stress-sensitive and one stress-tolerant), while the second factor corresponded to the combinations between water deficit and seed pre-treatment with stress attenuators (T1 = 0.0 MPa [control]; T2 = −0.2 MPa [water deficit]; T3 = −0.2 MPa + hydropriming [8 h]; T4 = −0.2 MPa + gibberellic acid [0.5 mM]; T5 = −0.2 MPa + salicylic acid [0.05 mM]; T6 = −0.2 MPa + hydrogen peroxide [10 mM]).

The concentration of each attenuator and the exposure period were determined in preliminary tests. Based on the imbibition curves of both cultivars, obtained using blotting paper, an 8-h hydropriming period was established, during which seed moisture content and radicle emergence time were uniform across the two cultivars. In this stage, in addition to the tests performed in Stage I, enzymatic complex activity in the seedlings was also evaluated. After biometric analyses, normal seedlings from each replicate were collected and used to obtain the extracts.

For hydrogen peroxide and lipid peroxidation (MDA) analyses, crude extracts were obtained from 200 mg of fresh matter per replicate, disintegrated in liquid nitrogen (−196 °C), and macerated for 1 min in 2 mL of trichloroacetic acid (TCA, 0.1%) containing approximately 20% polyvinylpolypyrrolidone (PVPP). After homogenization, the extract was transferred to Eppendorf tubes and centrifuged at 10,000 rpm, 4 °C, for 5 min.

For superoxide dismutase, catalase, and ascorbate peroxidase determinations, crude extracts were obtained from 500 mg of fresh matter per replicate, disintegrated in liquid nitrogen (−196 °C). After the addition of 20% PVPP, the sample was homogenized and macerated for 1 min in 100 mM potassium phosphate buffer (pH 7.5), supplemented with 1 mM EDTA (ethylenediaminetetraacetic acid) and 3 mM DTT (dithiothreitol). The extracts were then transferred to Eppendorf tubes and centrifuged at 10,000 rpm for 30 min at 4 °C. All extraction procedures were conducted in an ice bath.

The following determinations were performed:

Hydrogen peroxide (H_2_O_2_): determined according to Alexieva et al. [40] using potassium iodide and spectrophotometric readings at 390 nm; results expressed as µmol H_2_O_2_ g^−1^ fresh mass.

Lipid peroxidation (MDA): determined according to Heath and Packer [41], based on malondialdehyde (MDA) production, a reactive metabolite that reacts with thiobarbituric acid (TBA). Absorbance was measured at 535 and 600 nm; results expressed as µmol MDA g^−1^ fresh mass.

All enzymatic activities were normalized by the total protein concentration previously determined in the extract and expressed in units per mg of protein.

Superoxide dismutase activity (SOD): quantified according to Gianopolitis and Ries [42] under white light (15 W fluorescent lamp) for 5 min. Aliquots of 50 μL extract were transferred to a reaction medium containing 85 mM sodium phosphate buffer (pH 7.8), 780 μL methionine (13 mM), 30 μL EDTA (0.1 mM), and 225 μL NBT (75 mM). The reaction was initiated by adding 150 μL riboflavin (5 μM), in a final volume of 3 mL. Absorbance was measured at 560 nm; results expressed as enzyme units per minute per gram of fresh mass (U SOD mg^−1^ protein).

Catalase activity (CAT): determined by monitoring H_2_O_2_ degradation spectrophotometrically at 240 nm for 1 min [43], with modifications by Azevedo et al. [44]. The reaction medium contained 1 mL of 100 mM potassium phosphate buffer (pH 7.5) and 25 μL of 1 mM H_2_O_2_. The reaction was initiated by adding 25 μL of protein extract. Activity was calculated based on H_2_O_2_ decomposition over 60 s at 25 °C; results expressed as µmol min^−1^ mg^−1^ protein.

Ascorbate peroxidase activity (APX): determined according to Nakano and Asada [45]. Readings were taken over 1 min at 290 nm, using a molar extinction coefficient of 2.8 mmol^−1^ L cm^−1^. The 1 mL reaction medium consisted of 650 μL of 80 mM potassium phosphate buffer (pH 7.0), 100 μL ascorbate (5 mM), 100 μL EDTA, 100 μL H_2_O_2_, and 50 μL protein extract. Results were expressed as µmol min^−1^ mg^−1^ protein.

### 4.3. Statistical Analysis

Data were subjected to ANOVA, and when significant, means were compared using the Scott–Knott test (*p* ≤ 0.05) for cultivar and water deficit effects in Stage I. Subsequently, data were subjected to hierarchical cluster analysis by Ward’s minimum variance method, using Euclidean distance as the dissimilarity measure, with the freeware PAST 4. In Stage II, when the F-test was significant, cultivar means were compared using the *t*-test, and attenuator means using Tukey’s test, both at the 5% significance level. Statistical analyses were performed using the SISVAR^®^ 5.6 software [46].

## 5. Conclusions

Water deficit impairs germination and seedling vigor in watermelon, regardless of cultivar tolerance level.

Pre-germinative seed treatments in cv. Crimson Sweet enhance tolerance through the stimulation of antioxidant enzyme activity, with GA being the most effective attenuator.

In cv. Fairfax, the primary defense mechanism against water deficit is the accumulation of compatible solutes, which reduces the intensity of enzymatic responses.

## Figures and Tables

**Table 1:** Germination (G), germination speed index (GSI), shoot length (SL), root length (RL), shoot dry mass (SDM), root dry mass (RDM), total soluble sugars (TSS), free amino acids (FAA), and free proline (PRO) of watermelon cultivars under different water deficit levels.

Cultivrs	Stress	G	GSI	SL	RL	SDM	RDM	TSS	FAA	PRO
	MPa	(%)		(cm seedling^−1^)		(mg seedling^−1^)		mg/g FM	umol/g FM	
Crimson Sweet	0	98 aA	16.4 aB	8.3 aC	10.4 aB	7.1 aC	3.5 aD	1.2 cA	47.0 bB	0.1 cA
	−0.1	93 aA	14.4 bC	4.0 bB	8.8 aB	6.6 aC	4.2 aB	10.7 bD	90.0 aA	1.8 bD
	−0.2	80 bB	9.2 cC	1.7 cA	5.5 bB	4.4 bB	2.4 bD	19.5 aB	103.7 aC	2.8 aC
Fairfax	0	98 aA	22.7 aA	9.1 aB	13.9 aA	13.3 aB	8.1 aA	1.9 cA	61 cA	0.2 cA
	−0.1	98 aA	17.3 bA	3.8 bB	10.0 bB	11.5 bA	7.2 bA	15.0 bB	95 bA	2.4 bB
	−0.2	87 bA	12.2 cA	1.4 cA	7.6 cA	6.7 cA	6.3 bA	25.2 aA	120 aA	4.1 aA
Charleston Gray	0	100 aA	23.4 aA	9.9 aA	7.8 bC	12.8 aB	5.0 bC	1.8 cA	58.6 bA	0.1 cA
	−0.1	90 bA	15.7 bB	5.0 bA	12.0 aA	11.7 aA	6.2 aA	13.0 bC	92.4 aA	2.8 bA
	−0.2	82 cB	10.0 cC	2.4 cA	8.4 bA	6.5 bA	4.3 bC	18.6 aB	96 aC	3.4 aB
Charleston Super	0	99 aA	23.7 aA	9.8 aA	11.4 aB	15.2 aA	6.5 aB	1.4 cA	56.6 cA	0.1 cA
	−0.1	82 bB	15.3 bC	5.0 bA	10.1 aB	12.6 bA	6.3 aA	14.2 bB	95.6 bA	2.8 bA
	−0.2	61 cC	9.9 cC	1.4 cA	5.8 bB	5.4 cB	3.6 bC	19.4 aB	110 aB	4.2 aA
Omaru	0	99 aA	17.2 aB	10.6 aA	12.0 aB	12.1 aB	4.4 aD	1.3 cA	47.3 cB	0.1 cA
	−0.1	96 aA	16.0 bB	4.5 bA	11.4 aA	9.2 bB	4.3 aB	16.3 bA	90.9 bA	2.4 bB
	−0.2	78 bB	12.5 cA	1.9 cA	7.5 bA	5.9 cA	3.9 aC	19.7 aB	101.8 aC	4.1 aA
Congo	0	99 aA	17.5 aB	9.9 aA	11.0 aB	12.0 aB	5.4 bC	1.7 cA	50.6 bB	0.2 cA
	−0.1	96 aA	14.2 bC	5.0 bA	11.3 aA	9.2 bB	6.5 aA	13.7 bB	82.8 aB	2.2 bC
	−0.2	86 bA	11.0 cB	1.9 cA	7.4 bA	4.6 cB	4.9 bB	20.1 aB	86.6 aD	2.9 aC
CV (%)		5.3	4.7	10.4	11.9	11.3	12.5	10.5	6.3	13.1

Lowercase letters indicate differences among water restriction levels within each cultivar, while uppercase letters indicate differences among cultivars within each water restriction level, according to the Scott–Knott test at 5% probability.

## Data Availability

Data is contained within the article.
